# The Prevalence of Peer Sexual Harassment During Childhood in Australia

**DOI:** 10.1177/08862605241245368

**Published:** 2024-04-09

**Authors:** Gabrielle R. Hunt, Daryl J. Higgins, Megan L. Willis, Ben Mathews, David Lawrence, Franziska Meinck, Rosana Pacella, Hannah J. Thomas, James G. Scott, Holly E. Erskine, Eva Malacova, Divna M. Haslam

**Affiliations:** 1 Australian Catholic University, Banyo, QLD, Australia; 2 Australian Catholic University, Melbourne, VIC, Australia; 3 Australian Catholic University, Sydney, NSW, Australia; 4 Queensland University of Technology, Brisbane, Australia; 5 Johns Hopkins University, Bloomberg School of Public Health, MD, USA; 6 Curtin University, Perth, WA, Australia; 7 University of Edinburgh, UK; 8 North-West University, Vanderbijlpark, South Africa; 9 University of the Witwatersrand, Johannesburg, South Africa; 10 University of Greenwich, London, UK; 11 Queensland Centre for Mental Health Research, Wacol, Australia; 12 The University of Queensland, Brisbane, Australia; 13 QIMR Berghofer Medical Research Institute, Brisbane, Australia; 14 Children’s Health QLD, South Brisbane, Australia; 15 QIMR Berghofer, Medical Research Institute, Brisbane, QLD, Australia

**Keywords:** Sexual harassment, children, adolescents, gender, diversity, intersectionality, LGBTQIA+

## Abstract

Sexual harassment inflicted by adolescents on their peers is a major public health issue, but its prevalence across childhood is not known. We provide the first nationally representative data on the prevalence of peer sexual harassment across childhood, using cross-sectional data from the Australian Child Maltreatment Study (ACMS). The ACMS surveyed 8,503 people aged 16 and over about their experiences of child maltreatment and associated health outcomes. The prevalence of peer sexual harassment was assessed using the Juvenile Victimization Questionnaire (JVQ)-R2 Adapted Version (ACMS), with survey data weighted to reflect characteristics of the Australian population. Overall, 1 in 10 (10.4% (95% Confidence Intervals (CI) [9.7, 11.3])) Australians experienced peer sexual harassment during childhood. Peer sexual harassment is an issue disproportionately affecting gender-diverse individuals (24.0%, 95% CI [15.5, 35.2]) and women (15.3%, 95% CI [14.0, 16.7%]), compared to men (5.0%, 95% CI [4.3, 5.9]). Rates of peer sexual harassment were also very high among sexuality diverse participants (prevalence estimates ranging between 14.2% and 29.8%). Peer sexual harassment was predominately inflicted by male peers (9.6%, 95% CI [8.9, 10.4]), compared to 1.8% (95% CI [1.5, 2.2]) reporting harassment from female peers. These findings have implications for understanding and reducing attitudes supporting peer sexual harassment in childhood, particularly against girls and gender and sexuality diverse youth, and associations with other gendered violence both in childhood and later life.

The “Me Too” movement and numerous high-profile cases have highlighted the prevalence of sexual assault and violence, including sexual harassment (Brown et al., 2020). Sexual harassment is widespread across society, spanning the highest levels of public office and professions, including medicine (Fnais et al., 2014), social and workplace settings (Australian Bureau of Statistics, 2017), and secondary school settings (Vega-Gea et al., 2016). While defined in different ways in law, policy, and epidemiology, sexual harassment can generally be understood as involving unwanted conduct of a sexual nature which makes a person feel offended or intimidated (Australian Human Rights Commission, 2010; Mathews & Bismark, 2015; Vega-Gea et al., 2016). We adopt the definition used by the Australian Human Rights Commission (2010, para 1), stating that sexual harassment includes “any unwanted or uninvited sexual behavior that is offensive, embarrassing, intimidating or humiliating”. Sexual harassment can be conceptually distinguished from sexual assault or abuse, partly because of the different nature of the acts typically constituting it, and because the element of sexual gratification is present for assault and abuse but is not required for harassment (Mathews & Collin-Vézina, 2019).

Whether between adults or adolescents, sexual harassment is a gendered form of violence disproportionately perpetrated by men and boys against women and girls (Brown et al., 2020). Women and girls report higher rates of sexual harassment than men and boys and are also more likely to report sexual harassment by their opposite-gender peers (Australian Bureau of Statistics, 2017; Espelage et al., 2016; Petersen & Hyde, 2009). By contrast, boys are more likely to report sexual harassment by same-gendered peers (Clear et al., 2014; Lichty & Campbell, 2012). Although some studies have reported similar victimization rates between boys and girls (Lei et al., 2020; McMaster et al., 2002; Mumford et al., 2013), these are the exception. Recent findings have also pointed to the disproportionate experiences of sexual harassment and other forms of violence for gender- and sexuality-diverse youth (Gill & McQuillan, 2022; Valido et al., 2022).

Peer sexual harassment is often normalized, and tolerated (McMaster et al., 2002), which inadvertently increases the risk for further victimization both in childhood and in later life. It is associated with an increased risk of serious adverse outcomes such as self-harm and suicidal thoughts (Bucchianeri et al., 2014; Chiodo et al., 2009), poor self-esteem (Duffy et al., 2004; Lindberg et al., 2007), and other physical and psychological health problems (Duffy et al., 2004; Gruber & Fineran, 2008; Sagrestano et al., 2019; Skoog & Kapetanovic, 2021). Despite its importance, the literature indicates several issues limiting our understanding of the prevalence of peer sexual harassment. Different approaches in survey instrumentation, definition, and measurement have produced considerable variance in prevalence estimates (Conroy, 2013; Eom et al., 2014; Lei et al., 2020). Studies have used a variety of measures, different age groups, and inconsistent time parameters when measuring the prevalence of peer sexual harassment (Lei et al., 2020; Vega-Gea et al., 2016). Schools and other youth-serving organizations have historically dealt with peer sexual harassment in the broader context of peer bullying, which has led to less attention being directed to this unique issue and conflation of these two forms of victimization (Eom et al., 2014; Lei et al., 2020; Shute et al., 2016). Peer bullying has been historically understood as behaviors repeated over time where there is an imbalance of power or strength (Olweus, 2010). However, young people do not always conceptualize bullying as requiring repeated behaviors or the presence of a power imbalance. Other researchers have suggested bullying should be understood to include an intent to hurt, the experience of harm, or the likelihood of the bullying being repeated (Cuadrado-Gordillo, 2012; Hellström et al., 2015). While some instances of peer sexual harassment may meet the criteria for peer bullying, it is important to distinguish between these two concepts to ensure accurate prevalence estimates and more targeted prevention and response strategies (Hill & Kearl, 2011).

Both in Australia and internationally, there is limited reliable evidence from nationally representative samples about the prevalence of peer sexual harassment between children or adolescents aged under 18 across the span of childhood. To date, prevalence estimates of peer sexual harassment have typically focused on surveying adolescents in school settings. The first large, representative estimates in the United States (U.S.) showed that 85% of girls and 76% of boys reported being sexually harassed by a peer at school (American Association of University Women Educational Foundation, 1993). More recent estimates in the U.S. have reported that nearly half of school students surveyed had experienced some form of sexual harassment (40%–48%, respectively; Crowley & Cornell, 2020; Hill & Kearl, 2011). Others have reported prevalence rates as high as 95% for girls and 88% for boys (Ormerod et al., 2008).

In Australia, the most detailed evidence to date is from a study of 4,098 participants (1,938 boys and 2,160 girls) aged 11 to 19 (*M* = 15) in secondary schools in New South Wales (Lei et al., 2020). This study administered a nine-item modified version of the instrument used in McMaster et al. (2002), assessing experiences in the previous school term of incidents that were specified as “unwanted,” but not as “offensive” or “intimidating.” Lei et al. (2020) found an overall prevalence of 41.2% (42.5% for boys and 40.0% for girls). The most frequently reported experience was “sexual comments, jokes, gestures, or looks” reported by 32.2% of girls and 29.9% of boys. Participants were also asked about a range of experiences, including indecent exposure (“flashing their private parts at you”) and acts that may constitute sexual assault or sexual abuse (“poking, grabbing, or pinching you in a sexual way”). This study found inverted prevalence trends, with boys more frequently reporting multiple types of experiences than girls (Lei et al., 2020). However, their prevalence estimates were not specific to those inflicted by peers as participants may have reported on experiences of sexual harassment by any person in the previous term, including adults. Similarly, the broad measurement of “unwanted” sexual acts may have inflated these prevalence estimates. It is likely that many people experience “unwanted” sexual behaviors, but this does not mean that they are also experienced as offensive, intimidating, or humiliating, as defined by the Australian Human Rights Commission (2010).

Other studies have also included incidents of rape and other contact sexual abuse in their measurement of peer sexual harassment (e.g., “forced you to do something sexual”) (American Association of University Women Educational Foundation, 1993; Conroy, 2013; Timmerman, 2003; Valido et al., 2022; Valik et al., 2022). The inclusion of items that assess behaviors that may be more properly understood as sexual assault or sexual abuse suggests some prevalence estimates may be inflated by including the total experience of all types of sexual assault and violence by peers.

Accordingly, peer sexual harassment prevalence estimates to date have been limited to small sample sizes or samples that are not nationally representative and do not cover the entire span of childhood. In addition, they typically have employed broad conceptualizations of peer sexual harassment. In Australia, estimates to date have also been limited to comparisons between cisgender males and females and have not explored the potential interplay between diverse gender or sexuality identities and the experience of peer sexual harassment. The aim of this study was to determine nationally representative prevalence estimates of peer sexual harassment across the entire span of childhood (any experience before the age of 18) using Australian Child Maltreatment Study (ACMS) data and a rigorous conceptual model of sexual harassment.

## Method

This study utilized data from the ACMS. The ACMS is a cross-sectional survey of 8,503 Australians aged 16 years and older. The ACMS included an oversample of young people aged 16 to 24 (*n* = 3,503), with the remaining sample having approximately 1,000 participants in each age category (25–34, 35–44, 45–54, 55–65, and 65 years or more; Table 1). Women made up just over half of the sample (50.9%; men = 48.1%; diverse genders = 1.0%; Haslam et al., 2023). The sample was compared to Australian Census data and to National Health data to ensure it was representative of the population, including across factors such as gender, age, and Indigenous status, and across all states and territories. Participants of our study were slightly more likely to have higher income and education, socioeconomic advantage, and be Australian-born. This was addressed by applying population weights in the analysis to ensure outcomes were representative of the general population (Haslam et al., 2023).

**Table 1.** Weighted Prevalence Estimates (with 95% CIs) of Peer Sexual Harassment During Childhood, by Age Group and Gender.

Age Group	Number of Respondents *n*	Experienced Peer Sexual Harassment (%)	[95% CI]
All ages	8,503	10.4	[9.7, 11.3]
Men	4,195	5.0	[4.3, 5.9]
Women	4,182	**15.3**	[14.0, 16.7]
Diverse genders	126	**24.0**	[15.5, 35.2]
16–24 years	3,500	13.5	[12.3, 14.8]
Men	1,748	4.8%	[3.8, 5.9]
Women	1,662	**21.2**	[19.1, 23.4]
Diverse genders	90	**35.9**	[26.0, 47.0]
25–34 years	1,000	13.3	[11.2, 15.8]
Men	516	6.5	[4.5, 9.4]
Women	470	**20.0**	[16.5, 24.1]
35–44 years	1,000	10.7	[8.9, 12.9]
Men	476	6.0	[4.2, 8.5]
Women	516	**15.4**	[12.3, 19.1]
45–54 years	1,002	10.2	[8.4, 12.4]
Men	477	5.8	[3.9, 8.5]
Women	521	**14.6**	[11.6, 18.2]
55–64 years	1,001	9.0	[7.3, 11.1]
Men	487	3.2	[2.0, 5.1]
Women	509	**14.3**	[11.2, 17.9]
65 years and older	1,000	6.9	[5.4, 8.8]
Men	491	3.6	[2.2, 5.9]
Women	504	**9.4**	[7.1, 12.5]

*Note*. Confidence Intervals (CIs) for women or participants with diverse genders that do not overlap with men are in bold (statistically significant at the 5% level). Diverse genders of older cohorts were not reported due to small sample sizes.

The ACMS was funded and designed as a survey of the general population. Therefore, we determined that it would not be ethically or methodologically appropriate to separately collate, analyze, or present data obtained from Aboriginal or Torres Strait Islander participants. This decision was confirmed through independent and external consultation.

Participants were interviewed about their experiences of child maltreatment prior to the age of 18, as well as other adverse experiences and health outcomes. The JVQ-R2: Adapted Version (ACMS) was used to assess experiences of child maltreatment. This instrument was found to be reliable and valid for measuring child maltreatment (Mathews, Meinck et al., 2023).

### Sexual harassment

We adopt the language and conceptualization of generalized sexual harassment used in the ACMS (Mathews & Collin-Vézina, 2019; Mathews, Meinck et al., 2023). In the ACMS, generalized sexual harassment was measured by participants’ responses to the item asking: “Did anyone ever say, write or do something sexual to you that was offensive or intimidating?.” The ACMS instrument adaptation process modified the JVQ and refined the terminology for the sexual harassment item from “hurt your feelings” to “offensive or intimidating” to align with nationally recognized definitions of sexual harassment in policy settings (Australian Bureau of Statistics, 2017; Australian Human Rights Commission, 2010) and legal settings (Mathews & Bismark, 2015). This is a similar conceptualization to the legal concept of “sexual harassment,” which was developed to underpin legal protections for women in workplaces against discriminatory sexual behaviors, creating duties for employers and employees to protect women’s rights to equal opportunity in the workplace and a safe work environment (Mathews & Bismark, 2015). More broadly, in a sense spanning both legal and epidemiological approaches, sexual harassment can be understood as the experience of unwelcome sexual conduct committed in circumstances where a reasonable person would have anticipated the harassed person would be offended, humiliated, or intimidated (Mathews & Bismark, 2015). A further example of this general approach can be seen in Australia’s Personal Safety Survey (Australian Bureau of Statistics, 2017), which defined “sexual harassment” as experiencing or being subjected to behaviors that the person found improper or unwanted that made them feel uncomfortable, and/or was offensive due to their sexual nature. This aligns with the approaches employed in several rigorous studies of peer sexual harassment, which conceptualize sexual harassment as “an unwanted and unwelcome sexual behavior” (Ortega et al., 2010, p. 248), “[that] cause[s] distress and discomfort to the victims, which can interfere with the normal life of students in schools” (Vega-Gea et al., 2016, p. 48). Although some studies of peer sexual harassment do include more serious acts of physical violence even when using this concept (Ortega et al., 2010; Vega-Gea et al., 2016), in general, comprehensive studies of sexual abuse or sexual assault and sexual harassment, should distinguish between these different levels of gravity.

### Sexual Abuse

The ACMS captured other experiences of sexual violence, namely, non-contact and contact sexual abuse, in four separate items designed to replicate the original JVQ and to conform with a robust conceptual model of “child sexual abuse” (Mathews & Collin-Vézina, 2019). Non-contact sexual abuse includes abusive exposure or voyeurism for sexual gratification, constituted by someone looking at a child’s private parts when they should not have or making a child look at their private parts. Contact sexual abuse includes touching a child’s private parts for sexual gratification or forcing the child to touch the offender’s private parts, attempted forced sexual intercourse, and completed forced sexual intercourse (Mathews, Meinck et al., 2023). These four items were understood to represent manifestations of child sexual abuse, and prevalence estimates for each of these experiences and for child sexual abuse as a whole are reported elsewhere (Mathews, Pacella et al., 2023).

The ACMS conceptualization of sexual harassment is a conservative approach as it does not include any items that would be more accurately defined as child sexual abuse (e.g., non-contact and contact sexual abuse). Instead, we have focused specifically on instances of offensive or intimidating sexual language or behaviors, which can be conservatively understood as constituting sexual harassment while not necessarily always satisfying the conceptual model of child sexual abuse, for example, by lacking the element of sexual gratification (Mathews & Collin-Vézina, 2019). The differentiation of peer sexual harassment from child sexual abuse and peer victimization is important as it has the advantage of providing a more accurate estimate of the prevalence and nature of peer sexual harassment, as well as informing an understanding of its association with other experiences including sexual abuse, and associated outcomes (Mathews, Pacella et al., 2023).

Accordingly, this analysis focused on responses to the sexual harassment question, “Did anyone ever say, write or do something sexual to you that was offensive or intimidating?.” The ACMS collected data about a number of different perpetrators, including adults, but in this study, we focus specifically on instances of sexual harassment inflicted by other children and adolescents aged under 18, understood here as peer sexual harassment. This included siblings (such as brothers, sisters, or other children or adolescents living in the home), romantic partners (including current or previous romantic partners during childhood), known peers (i.e., other children or adolescents known to the participant), and unknown peers (i.e., other children or adolescents that were unknown to the participant).

Data were also collected about participants’ gender and sexual identity. Diverse genders included those who are not cisgender, including trans and non-binary people. Sexual identities included heterosexual or straight, gay or lesbian, bisexual, and other. Due to small sample sizes, “other” sexual identities combined participants who identified as queer, asexual, pansexual, preferred not to have a label, other, refused, or don’t know. Further details about the methodology of the ACMS and the sample have been provided elsewhere (see Haslam et al., 2023).

### Statistical Analysis

Adopting methods used in the previous ACMS analyses, prevalence estimates were calculated for the whole sample and by gender, sexuality, and age group. Prevalence estimates were weighted by gender, age group, Indigenous status, country of birth, highest educational level, and residential socioeconomic status (Mathews, Pacella et al., 2023; Haslam et al., 2023). Analysis of different subtypes of peers was also completed, including siblings (brothers, sisters, or other children or adolescents living in the home), romantic partners (current or previous romantic partners in childhood), known peers (children or adolescents that were not siblings or romantic partners but were known to the participant), unknown peers (children or adolescents not known to the participant), and comparison of male and female peers.

Prevalence of peer sexual harassment was based on affirmative responses to the sexual harassment question, regardless of chronicity (Mathews, Pacella et al., 2023). We used crosstabulations with 95% Confidence Intervals (CIs) to calculate Australian population-weighted estimates of prevalence and to examine differences between subgroups such as age, gender, and sexuality. These were calculated as per the Taylor Series expansion method (Wolter, 2007) and were summarized as percentages (Haslam et al., 2023; Mathews, Pacella et al., 2023). In this method, CIs that do not overlap are considered statistically significant at the 5% level and have been marked in bold in the Tables. Analyses were conducted in IBM SPSS (Version 29) and were independently checked.

### Ethics Approval

The Australian Catholic University Human Research Ethics Committee approved the analysis of the ACMS dataset (2023-3004N). The original project was approved by the Queensland University of Technology Human Research Ethics Committee (#1900000477).

## Results

One in ten reported having been sexually harassed by a peer during childhood (10.4%, 95% CI [9.7, 11.3]; Table 1). Women across all age groups reported higher rates of having experienced peer sexual harassment (9.4%–21.2%) in childhood compared to men (3.2%–6.5%). Diverse genders also reported higher rates of having experienced peer sexual harassment (24.0%) than men and women in the youngest cohort (16–24 years, 35.9%). Prevalence rates of older cohorts of diverse genders were not reported due to low total participants in those age groups reporting gender diversity. The two youngest cohorts reported higher rates of experiencing peer sexual harassment during childhood (16–24 years: 13.5%; and 25–34 years: 13.3%) compared to the oldest cohorts (55–64 years: 9.0%; and 65 years and older: 6.9%).

Consistent with the above overall prevalence estimates of peer sexual harassment, women and participants of diverse genders reported higher rates of having experienced peer sexual harassment by each child or adolescent type compared to men (Table 2). Peer sexual harassment was most commonly inflicted by peers known to the participant (7.9%) but who were not siblings or in current or previous romantic relationships during childhood. This pattern was observed regardless of the gender of the participant (harassment from known peers was reported by 4.1% of men, 11.2% of women, and 16.6% of people with diverse genders). Overall rates of peer sexual harassment inflicted by siblings (1.4%), current or previous romantic partners (0.9%), or unknown children or adolescents (1.3%) were significantly lower. People with diverse genders reported substantially higher rates of experiencing peer sexual harassment by unknown peers (12.2%) compared to women (1.9%) or men (0.5%).

**Table 2.** Weighted Prevalence Estimates (with 95% CIs) of Peer Sexual Harassment During Childhood, by Gender and Relationship to the Peer Perpetrator.

Gender	Sexual Harassment From			
	Siblings	Romantic Partners	Known Peers	Unknown Peers
All	1.4% [1.1, 1.7]	0.9% [0.7, 1.1]	7.9% [7.2, 8.6]	1.3% [1.1, 1.6]
Men	0.6% [0.4, 1.0]	0.1% [0, 0.3]	4.1% [3.4, 4.9]	0.5% [0.3, 0.8]
Women	2.1% [1.6, 2.7]	1.5% [1.1, 1.9]	11.2% [10.1, 12.4]	1.9% [1.5, 2.4]
Diverse genders	*	4.1% [2.1, 7.7]	16.6% [9.7, 26.9]	12.2% [5.6, 24.2]

*Note.* Siblings included brothers, sisters, or other children or adolescents who lived with the participant during childhood. Romantic relationships included current or previous romantic partners at the time of the peer sexual harassment. Peers included children and adolescents who were known to the participant but were not siblings or in romantic relationships with the participant.

* Not reported as fewer than five sample participants indicated sexual harassment by a sibling.

Peer sexual harassment was most commonly inflicted by male peers (9.6%), particularly those known to the participant (8.9%; Table 3). Participants with diverse genders reported the highest rates of having experienced peer sexual harassment by both male (22.8%) and female (8.0%) peers, compared to men (4.3% for male peers, 1.4% for female peers) and women (14.5% for male peers, 2% for female peers).

**Table 3.** Weighted Prevalence Estimates (with 95% CIs) of Peer Sexual Harassment During Childhood, by Gender and Peer Perpetrator.

Gender	Number of Respondents *n*	Sexual Harassment by Any Male Peer	Sexual Harassment by Known Male Peer	Sexual Harassment by Any Female Peer	Sexual Harassment by Known Female Peer
All ages	8,503	9.6% [8.9–10.4]	8.9% [8.2–9.7]	1.8% [1.5–2.2]	1.7% [1.4–2.0]
Men	4,195	4.3% [3.6–5.1]	4.0% [3.3–4.8]	1.4% [1.1–1.9]	1.3% [1.0–1.7]
Women	4,182	14.5% [13.2–15.8]	13.3% [12.1–14.6]	2.0% [1.6–2.7]	1.9% [1.5–2.5]
Diverse genders	126	22.8% [14.5–34.0]	19.0% [11.8–29.3]	8.0% [3.4–17.8]	8.0% [3.4–17.8]

*Note.* Male peers included any male children or adolescents (siblings, romantic partners, other male peers), including those unknown to the participant. Female peers included any female children or adolescents (siblings, romantic partners, other female peers), including those unknown to the participant. Known males included brothers or other male children living in the home, current or previous boyfriends, or other known male peers. Known females included sisters or other female children living in the home, current or previous girlfriends, or other known female peers.

Although men had lower rates of having experienced peer sexual harassment in total (5%; Table 1), men with diverse sexualities reported higher rates of peer sexual harassment than heterosexual/straight men (Table 4). For example, men who identified as bisexual (17.3%) and other sexualities (14.2%) reported higher rates of experiencing peer sexual harassment than heterosexual (4.4%) or gay men (8.9%) during childhood. Women with diverse sexualities also reported higher rates of experiencing peer sexual harassment – including those identifying as bisexual (29.4%), lesbian (23.8%), and other sexualities (20.1%), compared to heterosexual women (14.2%). Those with diverse genders who identified as heterosexual or straight (17.5%) and other sexualities (29.8%) also reported high rates of having experienced peer sexual harassment in childhood.

**Table 4.** Weighted Prevalence Estimates (with 95% CIs) of Peer Sexual Harassment During Childhood, by Sexuality and Gender.

Sexuality	Number of Respondents	Experienced Peer Sexual Harassment (%)	[95% CIs]
Heterosexual or straight			
Men	3,830	4.4	[3.7, 5.3]
Women	3,570	**14.2**	[12.9, 15.7]
Diverse genders	20	**17.5**	[5.7, 43.0]
Gay or lesbian			
Men	128	8.9	[4.5, 16.6]
Women	68	23.8	[12.3, 41.2]
Diverse genders	11	5.2	[0.6, 31.8]
Bisexual			
Men	126	17.3	[9.5, 29.3]
Women	319	29.4	[23.4, 36.3]
Diverse genders	27	29.3	[14.5, 50.3]
Other sexualities			
Men	111	14.2	[7.6, 25.0]
Women	225	20.1	[14.4, 27.3]
Diverse genders	68	29.8	[16.9, 46.9]

Confidence Intervals (CIs) for women or participants with diverse genders that do not overlap with men are in bold (statistically significant at the 5% level). Due to small sample sizes, “other” sexual identities included participants who identified as queer, asexual, pansexual, preferred not to have a label, other, refused, or don’t know.

Analysis by gender of the peer showed that peer sexual harassment is mostly inflicted by male children or adolescents and is more likely to have been experienced by women and diverse genders than by men during their childhood (Table 5).

**Table 5.** Weighted Prevalence Estimates (with 95% CIs) of Sexual Harassment by A Male or Female Peer During Childhood, by Sexuality and Gender.

Sexuality	Number of Respondents	Total Sexual Harassment by Any Male Peer (%)	[95% CIs]	Total Sexual Harassment by Any Female Peer (%)	[95% CIs]
Heterosexual or straight					
Men	3,830	3.8	[3.1, 4.6]	1.2	[0.8, 1.6]
Women	3,570	**13.4**	[12.1, 14.8]	1.6	[1.1, 2.2]
Diverse genders	20	**17.5**	[5.7, 43.0]	4.3	[0.8, 19.5]
Gay or lesbian					
Men	128	8.6	[4.3, 16.4]	2.5	[0.7, 8.8]
Women	68	23.8	[12.3, 41.2]	4.2	[1.1, 14.9]
Diverse genders	11	5.2	[0.6, 31.8]	*	*
Bisexual					
Men	126	15.1	[7.8, 27.3]	4.7	[2.0, 10.7]
Women	319	28.3	[22.3, 35.2]	6.5	[3.9, 10.7]
Diverse genders	27	23.6	[11.0, 43.5]	7.2	[2.0, 23.0]
Other sexualities					
Men	111	9.4	[4.1, 19.9]	7.6	[3.3, 16.6]
Women	225	19.0	[13.4, 26.1]	5.3	[2.5, 10.6]
Diverse genders	68	29.0	[16.2, 46.2]	12.1	[3.9, 31.9]

*Note.* Confidence Intervals (CIs) for women or participants with diverse genders that do not overlap with men are in bold (statistically significant at the 5% level). Male peers included any male child or adolescent (siblings, romantic partners, other male peers), including those unknown to the participant. Female peers included any female child or adolescent (siblings, romantic partners, other female peers), including those unknown to the participant. Due to small sample sizes, “other” sexual identities included participants who identified as queer, asexual, pansexual, preferred not to have a label, other, refused, or don’t know.

* not reported as fewer than five sample participants.

## Discussion

We provide the first nationally representative prevalence estimates of peer sexual harassment during childhood in Australia. Our analysis of ACMS data found peer sexual harassment is a prevalent issue in Australia, with one in ten participants reporting having experienced peer sexual harassment during childhood. Peer sexual harassment was more prevalent in the younger cohorts compared to those who were 55 years and older. We also found that a disproportionately high number of women and gender and sexuality-diverse people experienced sexual harassment by their peers during childhood. This is consistent with previous research that has shown peer sexual harassment during childhood was a gendered issue (Brown et al., 2020; Espelage et al., 2016; Fineran & Bolen, 2006; Petersen & Hyde, 2009). Peer sexual harassment was most commonly inflicted by male children and adolescents, particularly those known to the participant. This was true regardless of the gender identity or sexual orientation of the participant. Overall, the proportion of Australians who experienced peer sexual harassment by siblings (1.4%), romantic partners (0.9%), unknown adolescents (1.3%), or female peers (1.8%) was low.

In the youngest cohort aged 16 to 24 years, individuals with diverse gender identities reported the highest rates of experiencing peer sexual harassment during childhood. The oldest cohorts (55 years and older) reported a lower overall prevalence of peer sexual harassment. It is important to consider why rates of peer sexual harassment have increased, especially when we acknowledge other evidence indicating child sexual abuse by adolescent peers has also increased (Mathews et al., 2024). Factors explaining this might include greater cultural awareness of the inappropriateness of sexual harassment leading to increased recollection of victimization, reduced memory of childhood events in those who are older (Mills et al., 2016), the influence of media (Ward, 2016), or a true increase in the prevalence of peer sexual harassment.

Participants who identified as having diverse sexualities were more likely to have experienced peer sexual harassment in childhood. Explanations for this include cultural norms that support homophobic and other discriminatory attitudes as well as heteronormative cultural pressures (Brown et al., 2020) or higher rates of other types of victimization including peer bullying and sexual abuse (McGeough & Sterzing 2018; Schneeberger et al., 2014), thereby increasing the risk for experiencing further harm.

Overall prevalence rates were lower than those from previous studies, such as the study by Lei et al. (2020) of peer sexual harassment in Australian high schools. This disparity can be seen, for example, in our rates for the 16 to 24 year age group (13.3%, comprising 4.6% of men, 21.1% of women, and 34.8% of diverse genders), which contrast with those reported by Lei et al. (2020) (42.5% of boys and 40% of girls experiencing at least one incident of peer sexual harassment at school in the previous school term); the disparity for girls is clear, and that for boys is especially marked. This disparity may partly be explained by Lei et al.’s (2020) broader conceptualization of sexual harassment, which included some acts the ACMS treated as sexual abuse and did not count as sexual harassment. In addition, and likely more influential in producing different results, Lei et al. (2020) did not specify that the act had to be experienced as offensive or intimidating. Given the nature of some of the acts (e.g., showing or sending sexual pictures or images; making sexual comments or jokes or gestures), and the high prevalence of male endorsement of some of these acts (which contrasts with the typical male-on-female trend of offensive or intimidating sexual harassment), it is possible that a considerable proportion of the acts characterized as sexual harassment occurred in a manner that may not fit the model of sexual harassment requiring offense or intimidation.

In contrast, the current study conceptualized sexual harassment in the manner adopted by the ACMS, which was chosen in order to conform more closely with legal and social science literature; accordingly, sexual harassment was operationalized to include experiences of anything said, written or done of a sexual nature, that was offensive or intimidating (Mathews et al., 2021). Other experiences of sexual assault and violence, such as non-contact and contact sexual abuse and online victimization were captured separately in other items on the ACMS. It is possible that this conservative approach to estimates of peer sexual harassment produces an underestimate, although this approach was deemed to capture the respective prevalence of both sexual harassment and child sexual abuse more accurately in Australia. Similarly, the inclusion in an ACMS sexual harassment estimate of non-contact sexual abuse by peers would result in a smaller disparity between the two studies. Future studies may usefully capture a number of experiences in the manner adopted by Lei et al. (2020) while also specifying that the experience was offensive or intimidating.

With sexual harassment occurring both at the highest levels of office in Australia and also to our children and adolescents (Brown et al., 2020), we must consider cultural and political factors that reinforce sexism and the normalization of offensive and intimidating sexual language to girls and diverse individuals, in particular. In our society, peer sexual harassment is often normalized, and tolerated, seen as a normal part of a growing interest in sexual activity around the onset of puberty (McMaster et al., 2002). “Boys will be boys” attitudes normalize and downplay the seriousness of sexual harassment by peers (Brown et al., 2020; Sandler & Stonehill, 2005). It is clear that we need primary prevention messages and public health approaches targeting boys and addressing cultural norms around violence against women and girls and other youths with diverse genders and/or sexualities. It is imperative that we consider how we can protect girls, and LGBTQIA+ children and adolescents from experiences of sexual harassment by peers.

Global evidence on the prevention of violence against women and girls points to the effectiveness of rigorous and long-term, community-based advocacy programs aimed at shifting harmful gender attitudes, as well as school-based interventions aimed at addressing sexual violence and cultural norms around gender in relationships (Kerr-Wilson et al., 2020; World Health Organization, 2018). Adults in the community and in our institutions are key to the prevention of peer sexual harassment. Training, resources, and support are needed for adults on how to effectively respond to instances of peer sexual harassment. A public health approach is needed to address homophobic attitudes and other discriminatory beliefs, sexism, sexualized gender stereotypes, and cultural support for gender-based violence. In the Australian context, Indigenous-led research that addresses culturally safe approaches to research and practice would also enhance our knowledge and understanding of the prevention of peer sexual harassment.

### Limitations and Directions for Future Research

As the ACMS data was based on retrospective report, it is unclear when participants with a diverse identity may have become aware of their own gender or sexual identity and whether or when they may have revealed this to others. It is, therefore, not clear if their disproportionate experiences of peer sexual harassment were related to their reported gender or sexual identity at the time of the study. However, the over-representation of women and gender or sexually diverse individuals reporting sexual harassment by peers, particularly male peers, during childhood has significant implications. Similarly, women and gender and sexually diverse individuals also experience significantly higher rates of maltreatment during childhood (Higgins et al., 2024).

It is unlikely that significant numbers of respondents endorsed the sexual harassment item in reference to instances which met the criteria for the four child sexual abuse items or conflated such harassment experiences with proximate abusive experiences. We considered this potentiality in cognitive testing of the survey instrument in order to ensure that participants were able to differentiate as intended between their various experiences. Moreover, the construct of sexual harassment (especially its core concept of being “offensive or intimidating,” and the multiple terms used (and used sequentially: “say, write, or do”) in the screener used to measure it is so significantly different to the items for sexual abuse that we are confident in being able to rely on these separate results.

Although information relating to the context in which the sexual harassment occurred is not available with the ACMS data, we can assume that much of the sexual harassment by peers may have occurred at, or in the context of, relationships based on attendance at school, sporting clubs, youth groups, or in the community (Timmerman, 2003). We must consider the role these organizations and the adults in these organizations, particularly schools, can play in the prevention of peer sexual harassment. Findings from the Australian Human Rights Commission (2022) have also pointed to the prevalence of workplace-based sexual harassment for adolescents, with 60% of women and 25% of men aged 15 to 17 having been sexually harassed in the workplace in the previous 5 years. Further research is needed to explore the context in which sexual harassment occurs in childhood, including risk factors for both victimization and perpetration (Fineran & Bolen, 2006). Analysis of the impact that peer sexual harassment has on physical and mental health, independent of child sexual abuse or other experiences of child maltreatment, is also needed.

We need to examine how violence against women, girls, and individuals with diverse genders or sexualities has been normalized in our society, such that it also affects our children and adolescents (Brown et al., 2020). National and global conversations are needed at the legislative and policy level to understand what interventions and targets are needed to prevent sexual harassment at every age, but especially in childhood.
